# Robot-Assisted Laparoscopic Renal Schwannoma Excision

**DOI:** 10.1089/cren.2016.0111

**Published:** 2016-11-01

**Authors:** Jeremy Kelley, Ryan Collins, Christopher Allam

**Affiliations:** ^1^ Department of Urology, San Antonio Military Medical Center, JBSA Fort Sam Houston, Texas.; ^2^ Department of Pathology, San Antonio Military Medical Center, JBSA Fort Sam Houston, Texas.

**Keywords:** kidney, schwannoma, robot-assisted laparoscopy

## Abstract

***Background:*** To report the first case of a renal schwannoma excised with robot-assisted laparoscopy.

***Case Presentation:*** A 43-year-old Caucasian female patient with vague abdominal symptoms was noted to have incidental right renal mass. Physical examination and laboratory tests were within normal limits. CT revealed a 4.6 cm heterogeneous enhancing right renal mass arising near the hilum. RENAL nephrometry score was 11a. She was treated by right robot-assisted laparoscopic nephrectomy. She recovered well without complications. Pathology analysis revealed a benign renal schwannoma.

***Conclusion:*** Renal schwannoma is a rare kidney tumor. We report the first known case of this tumor excised by robot-assisted laparoscopic nephrectomy.

## Introduction and Background

Schwannomas are typically a benign tumor made up of Schwann cells arising from the peripheral nerve sheath. Historically, they most commonly involve the head, neck, and flexor surfaces of extremities, and surgical excision is the mainstay of treatment. Most of these tumors are sporadic; however, some are associated with neurofibromatosis type 2.^1^ Renal (or retroperitoneal) schwannomas are rare and were first described in 1955.^2^ Approximately 25 cases of renal schwannoma have been described in the literature. Renal schwannomas are predominately a benign tumor, but reports of malignant features have been reported. Iannaci et al. recently reported a case of epithelioid angiosarcoma arising within a renal schwannoma.^2^ Unfortunately, radiographic imaging of these tumors cannot differentiate between benign renal schwannomas and malignant tumors. Diagnosis of a renal schwannoma is usually made at time of pathologic examination. Radical nephrectomy is the treatment of choice because of the limited imaging characteristics and the possibility of malignant features within a renal schwannoma. Robot-assisted laparoscopic surgery is well established in excision of kidney tumors. To our knowledge, we present the first case of a benign renal schwannoma treated by robot-assisted laparoscopic nephrectomy.

## Presentation of Case

**FIG. 1.**
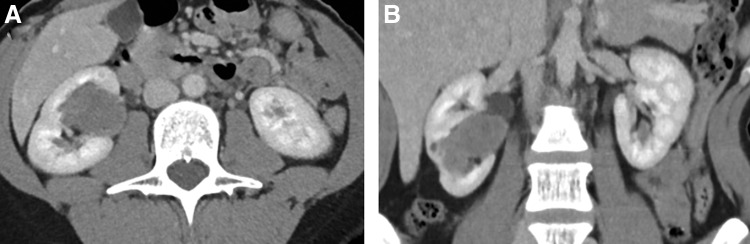
CT scan with axial **(A)** and coronal **(B)** views showing heterogeneously enhancing mass within the right kidney.

We present a 43-year-old Caucasian female with a few weeks history of vague, right-sided abdominal pain. She did not have any gastroesophageal symptoms. She denied any urologic symptoms including gross hematuria. She is a lifelong nonsmoker and did not have a family history of genitourinary cancer. Physical Examination was normal without palpable flank mass, pain, or evidence of sensorineural abnormalities. Her laboratory studies, which included a complete blood count, basic metabolic panel, coagulation studies, urinalysis, and human chorionic gonadotropin, were all within normal limits. She did previously have a history of hypercalcemia, but this had normalized at time of surgery. CT scan of the abdomen and pelvis with IV contrast was initially ordered and revealed a right heterogeneous kidney mass. A three-phase kidney CT scan revealed a 4.6 cm × 2.6 cm × 2.8 cm heterogeneously enhancing renal parapelvic mass without extension into the collecting system or renal vein (Fig. 1A). The right renal mass displayed increase in enhancement from 24.78 to 60.39 HU. The tumor was located centrally near the hilum and traversed into the lower pole of the kidney (Fig. 1B). RENAL nephrometry score was 11a. There was no lymphadenopathy or metastatic lesions noted on CT scan. A chest X-ray did not reveal any cardiopulmonary disease or nodules. She underwent a right robot-assisted laparoscopic nephrectomy (adrenal sparing). The surgery time was 149 minutes and blood volume loss was 50 mL. The patient was discharged home the following day. There were no postoperative complications.

**FIG. 2.**
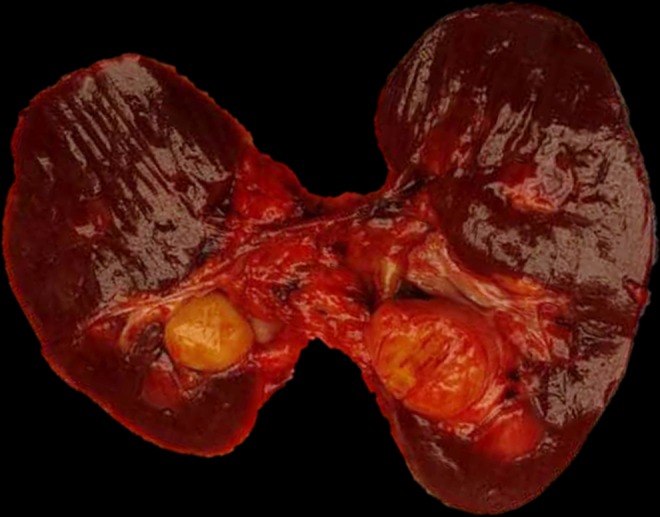
Gross examination demonstrates a well-circumscribed yellow tan nodular mass arising in the lower pole, near the hilum, of the right kidney.

**FIG. 3.**
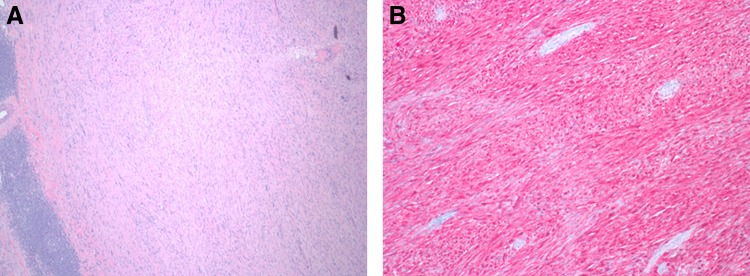
**(A)** Histologic examination shows a hypercellular spindled lesion with prominent peripheral lymphoid aggregates and scattered hyalinzed vessels (original magnification 40 × ). **(B)** An S100 immunohistochemical stain is strongly positive in the spindled cells (red chromogen, original magnification 100 × ).

Gross examination demonstrates a well circumscribed, nodular yellow lesion at the renal hilum, measuring up to 4.3 cm (Fig. 2). The nodule compresses the renal pelvis and bulges against the renal capsule, but does not grossly invade either structure. On histologic examination, the lesion is well circumscribed, with a thin capsule and no evidence of capsular invasion. The nodule is hypercellular, composed of numerous spindled cells arranged in a storiform pattern with mild nuclear pleomorphism and without prominent mitotic activity. There are numerous hyalinized vessels throughout, with scattered lymphoid aggregates lining the periphery (Fig. 3A). A panel of immunohistochemical stains was performed, which reveals that the tumor cells are strongly positive for S100 (Fig. 3B) and negative for smooth muscle actin, desmin, HMB-45, Mart-1, MiTF, and CD34. These results support a diagnosis of benign schwannoma.

## Discussion and Literature Review

Renal schwannomas are a rare entity comprising only 25 case reports in the English literature.^3^ Although the majority of these tumors are benign, malignant degeneration is possible. As with such a rare tumor, it is difficult to preoperatively diagnose renal schwannoma based on clinical presentation or radiographic imaging. Most of these tumors are discovered incidentally during work-up of vague abdominal symptoms. A review of renal schwannoma case reports revealed that flank pain (43%) and palpable mass (37%) were the most common presenting symptoms.^3^ Patient ages revealed a median of 48 years (14–84) with a slight female predominance. The majority of tumors are located in the parenchyma rather than the hilum.^3^ The average size of these tumors has been reported to be 10 cm,^2^ but analysis of cases since 2001 revealed a decrease in median size to 6.5 cm. This decrease in size is likely attributed to the increased utilization of CT imaging within the population.

Our patient was female, age 43, and tumor location was central parenchymal extending into lower pole of the right kidney. The radiographic characteristics revealed a heterogeneous enhancing mass that could easily be seen with renal cell carcinoma or oncocytoma. This CT scan finding is consistent with other renal schwannomas reported in the literature.^2^ Our patient did not have an MRI; however, it has been reported that MRI of these tumors reveals isointensity on T1-weighted images and high signal intensity on T2-weighted images. It has also shown on Gadolinium-enhanced T1-weighted images to reveal homogeneous enhancement in solid components of the tumor.^2^ Unfortunately this has not been shown to differentiate between benign and malignant renal schwannomas. Shun-Fa and colleagues. reported that 31% of renal schwannomas were malignant.^3^ Angiosarcomas have also been described to arise within renal schwannomas and these tumors are aggressive with direct tissue invasion, and metastasis is common.^3^ With the recent interest in renal mass biopsy, it is unclear whether the management of these lesions would change based on biopsy results. Owing to the limited nature of the biopsy sample, it would be difficult to rule out malignant transformation within a schwannoma, necessitating surgical removal for complete pathologic evaluation.

Because of the challenges of preoperatively diagnosing benign renal schwannomas, the mainstay of treatment has been nephrectomy. Patient presentation and radiographic imaging closely resemble renal cell carcinoma and, therefore, is treated as such. Historically, open radical nephrectomy was used for treatment. More recently, minimally invasive and nephron-sparing approaches have gained favor. The first reported laparoscopic excision of a retroperitoneal schwannoma tumors was described in the 1990s. Hanashima et al. reported laparoscopic tumor enucleation of a renal hilar tumor in a solitary kidney,^4^ which further described the feasibility of a minimally invasive approach to renal schwannomas. To our knowledge, our presented case is the first to incorporate the da Vinci^®^ surgical system for excision of renal schwannoma. This is the normal progression of surgical advancement into all kidney masses. Robot-assisted laparoscopy for partial nephrectomy (or nephrectomy) is standard of care for T1a renal tumors and is favored for T1b and T2 when feasible. Our patient had a centrally located 4.6 cm mass with RENAL nephrometry score of 11a. Although robotic partial nephrectomy would have been feasible on this complex tumor, our patient elected to have a radical nephrectomy. We used a four-arm configuration of the da Vinci robotic system during our nephrectomy. The patient was discharged home the day after surgery and no complications have been noted. The literature has shown the safety and feasibility of incorporating the da Vinci surgical system to the excision of a multitude of kidney tumors, and this case report broadens this application to renal schwannomas.

## Conclusion

Renal schwannomas are a rare entity as about 25 cases have been reported in the literature. These tumors can have a malignant potential. Renal schwannomas are difficult to diagnose based on patient presentation and imaging and often require a pathologic diagnosis. Robot-assisted laparoscopic excision is a safe and an effective treatment for these rare kidney tumors.

## Abbreviations Used

CT: computed tomography
HU: Hounsfield units

## References

[1] Pilavaki M, Chourmouzi D, Kiziridou A, et al. Imaging of peripheral nerve sheath tumors with pathologic correlation: Pictorial review. Eur J Radiol 2004;52:229 15544900 10.1016/j.ejrad.2003.12.001

[2] Iannaci G, Crispino M, Cifarelli P, et al. Epithelioid angiosarcoma arising in schwannoma of the kidney: Report of the first case and review of literature. World J Surg Oncol 2016;14:29 26842370 10.1186/s12957-016-0789-5 PMC4739400

[3] Shun-Fa H, Chung SD, Lai MK et al. Renal schwannoma: Case report and literature review. Urology 2008;72:716e3–716e6 10.1016/j.urology.2007.12.056 18314178

[4] Hanashima F, Yanaihara H, Hayashi T, et al. Laparoscopic non-clamping tumor enucleation of renal Hilum Schwannoma in a single kidney: A case report. Urol Case Rep 2015;3:211–214 26793556 10.1016/j.eucr.2015.07.012 PMC4714301

